# News and Notes

**Published:** 1900-02

**Authors:** 


### *NEWS AND NOTES.*

Dr. J. C. Harris died suddenly at his home in Suwanee, Ga., on December 31st, 1899. He was about 46 years of age.

Dr. C. A. Word, of Cedartown, was married in that city on January 10th, to Miss Carrie Houseal, of the same city.

Dr. Walter Lester Carr has assumed the editorial management of the *Archives of Pediatrics*, vice Dr. Floyd M. Crandall.

Drs. William Osler and Howard Kelly, of Baltimore, have been elected honorary members of the Royal Academy of Medicine in Ireland.

Dr. J. W. Bailey, of Gainesville, while in Florida recently, was bitten very severely by a dog. We trust that the doctor will soon be out again.

Dr. W. C. Jarnagin has been elected President of the Board of Health of Atlanta. Dr. B. W. Bizzell was also recently elected a member of this Board.

*The Raven*, edited by Dr. Ralcy H. Bell in St. Louis, Mo., is out with the first issue of Vol. V. It contains some terse sentiments. Dr. Bell formerly lived in Atlanta.

Dr. Wm. A. Hammond, a former Surgeon-General of the United States, died in Washington on January 5th. He was distinguished as a nerve specialist, and conducted a private sanatorium in the capital city.

SIR JAMES PAGET, the eminent English surgeon, died recently in England at a very advanced age. He has been living in retirement for several years, enjoying the rest to which his brilliant work as surgeon and teacher entitled him.

---

THE next International Congress of Tuberculosis will probably be held in London in the spring of 1901. A preliminary meeting for the purpose of making arrangements will shortly be held under the auspices of the British National Association for the Prevention of Tuberculosis.

---

PROFESSOR BIRCH-HIRSCHFELD, who was for many years professor of pathological anatomy in the University of Leipzig, Germany, is dead. He was looked upon as the most competent man on his line of work in all of Germany. He was especially kind to all American students.

---

WE have received a copy of the *Seaboard Medical and Surgical Journal,* published in Norfolk, Va. This is the first issue, and we congratulate its editor on the neat appearance of this copy. The editor, Dr. Lucien Lofton, is an old Atlanta boy, and we extend to him our best wishes.

---

THE Hartford (Conn.) Board of Health has decided to put shower baths in the public schools. Children who have been excluded from the schools on account of infectious disease in their homes will upon their return to school be required to take a bath and have their clothing disinfected.

---

DR. SCHENCK, who was professor in the University of Vienna, and sprang into fame through the promulgation of his ideas on sex-production, has been retired on demand from the Vienna Medical Faculty on account of his alleged "frivolous publication of scientific matter." News comes that he may settle in America.

THE University of Chicago has a new and unique branch in the Chicago Physiological School for the training of nervous and backward children. It is said to be the first of its kind in the world, and is intended as a home for boys and girls who are unable to cope with normal children owing to illness or infirmity.

---

THE *St. Louis Medical and Surgical Journal* is to be congratulated upon its January issue, in the shape of a brochure, by Albert S. Ashmead, M.D. We trust that the day is not far distant when Dr. Ashmead will be able to settle this vexed question, and will not be compelled to have a monthly discussion with himself.

---

MR. W. B. SAUNDERS, publisher, announces that the American Year-Book of Medicine and Surgery for 1900 will shortly appear in *two* volumes. The attention of the medical profession is invited to the new arrangement of this valuable publication by which may be obtained that portion in which one is specially interested.

---

IT is said that the adherents of cremation are rapidly increasing in Germany. Recent statistics place the number of cremation societies in the German Empire at thirty-seven, with an aggregate membership of thirty-seven thousand six hundred, an increase of membership of more than twenty-five thousand since the beginning of last year.

---

THE second annual meeting of the Tri-State Medical Association of the Carolinas and Virginia will be held in Charleston, S. C., during February of this year. This is one of the youngest of the Southern associations, but its meeting last year at Charlotte, N. C., gave evidence of its being one of the most scientific and practical organization of the kind in the country. The papers read at that meeting were all exceptionally able in character.

SCHLATTER'S famous case of extirpation of the stomach for cancer has at last reached a fatal issue. The patient left the hospital and lived for over a year after the operation. She died from a recurrence of the cancer. That a woman of this age should live and maintain her nutrition after the removal of the stomach is remarkable. She was over 65 years of age.

---

IT is most amusing to read some of the "queries" propounded in our "Home Medical Journals" like the *Alkaloidal Clinic*. Here is one:

Query 1017.—A woman can sit quietly on a chair, but on attempting to walk about the room her bowels begin acting.

This reminds us of the advertisement of "Cascarets" seen in the street-cars—"They work while you sleep"—certainly an unfortunate condition to be in.

---

THE newly organized medical club of Paris issues special cards of membership to out-of-town and foreign medical men visiting Paris. These entitle them to all the numerous privileges of the club during their stay. A special department has been organized to secure rooms for physicians during the exposition and supply information of all kinds. Dr. Doléris, 5, avenue de l'Opera, is the secretary. The club has the advantage of the quarters of the *Cercle National.*

---

THE president has made during his entire administration no better appointment than that of surgeon, now Gov. Wood as military governor of Cuba. It is no secret that the duties of Gov. Wood will be almost wholly of a civil character, and that he would have been appointed "Civil Governor" had it not been for the objections of the Cubans to the creation of such an office. Gov. Wood's administration of the province of Santiago has combined the scientific knowledge of the physician with the executive ability of the military leader and the statesman. Probably there is no American so popular and so largely trusted by the Cubans themselves as their new governor.—*Medical Times.*

---

THE Red Cross Society is virtually international, and in its efforts to alleviate the horrors of war is doing probably more than all the international congresses can do in a century. It is reported that the French Red Cross Society is preparing an expedition to the Transvaal. It has five field hospitals reserved for home needs, and is now trying to modify its charter so as to enable it to send these to foreign countries. The French Society for Aid to Wounded Soldiers has spent nearly $24,000 during the late Spanish war, and is now seeking funds to prepare four field hospitals for service—two for the British and two for the Boer wounded. An expedition is also being fitted out by the Russian Red Cross Society.

---

SUWANEE, GA., a town of 400 inhabitants, has adopted a novel way of obtaining a physician for the town. Not long ago the old resident physician died, so that a meeting of the leading citizens was held, at which they decided to advertise for a physician, and from the applicants this committee should make a selection. It seems as if this was done, and applications came in all the way from New York to Texas. Finally, out of the large number, Dr. O. B. Tucker was selected. In the Atlanta *Constitution* of recent date the following biographical sketch was telegraphed to that paper by the Suwanee correspondent :

He is the son of Dr. W. B. Tucker, of Chipley, Ga. He is a young man of unusually prepossessing appearance. His hair is kept parted with unerring precision from a point just over his nose straight back to where fashion has dictated that parts must stop. His hair is long, and graceful locks that would naturally fall over his ears are given an upward twist and protrude on either side on a level with his eyes.

It is unnecessary to deal further with the personal appearance of Dr.

Tucker. Suffice it to say that the remainder of his appearance is equally as striking as his head.

Dr. Tucker is an interesting conversationalist, although he dislikes talking about himself. He did, however, say in regard to his application for the position at Suwanee:

"I made application for the position here because I felt that I was competent. I took a course at the Atlanta Medical College during 1897 and 1898, but did not attempt to graduate from that institution. During the latter part of 1898 and the first part of 1899 I attended the medical department of the University of Georgia at Augusta.

"From there I went to the University of the South at Sewanee, Tenn., from which I graduated on January 1, 1900. I was on the staff of the Sewanee Charity Hospital, and have dealt with all sorts and manner of diseases."

The age of Dr. Tucker is still in doubt, as he has refused to announce just how old he is. Among the residents of Suwanee the guesses range from twenty-six to thirty years, and it is said that bets have been made, although they were declared off because of the reticence of the young physician.

In appearance he is about twenty or twenty-one years of age, although in learning he is doubtless much older. In reply to a question in regard to his age Dr. Tucker said:

"It is best, I think, when any one asks me my age, to give an evasive reply, because they might not be satisfied and there is so much prejudice against young physicians these days. If I keep silent they will have no cause to complain and we will be better off all around."

Dr. Tucker is already a prominent man in Suwanee. He is now known to the leading citizens of that place as "Doc," which is a name given only to those who hold the confidence of their fellows. He is going to be a member of the city council; there is no doubt of that. His name has already been mentioned, and it is understood that it is meeting with approval on all sides.

---

PRELIMINARY program of the fifth meeting of the Congress of American Physicians and Surgeons, to be held in Washington, D. C., May 1st, 2d and 3d, 1900.

President.—Prof. Henry P. Bowditch, M.D., LL.D., D.Sc., Boston, Mass.

Vice-presidents, ex officio.— The President of the American

Neurological Association, Dr. Edward D. Fisher, New York City; the President of the American Gynecological Society, Dr. George J. Engelmann, Boston, Mass.; the President of the American Dermatological Association, Dr. Henry W. Stelwagon, Philadelphia, Pa.; the President of the American Laryngological Association, Dr. Samuel Johnston, Baltimore, Md.; the President of the American Surgical Association, Dr. Robert F. Weir, New York City; the President of the American Climatological Association, Dr. Abraham Jacobi, New York City; the President of the Association of American Physicians, Dr. Edward G. Janeway, New York City; the President of the American Association of Genito-Urinary Surgeons, Dr. James Bell, Montreal, Canada; the President of the American Orthopedic Association, Dr. Harry M. Sherman, San Francisco, Cal.; the President of the American Physiological Society, Russell H. Chittenden, Ph.D., New Haven, Conn.; the President of the Association of American Anatomists, Dr. Burt G. Wilder, Ithaca, New York; the President of the American Pediatric Society, Dr. Henry Koplik, New York City; the President of the American Ophthalmological Society, Dr. Oliver F. Wadsworth, Boston, Mass.; the President of the American Otological Society, Dr. Henry G. Miller, Providence, R. I.;

Chairman of the Executive Committee—Landon Carter Gray, M.D., New York City.

Treasurer—Newton M. Shaffer, M.D., New York City.

Secretary—William H. Carmalt, M.D., New Haven, Conn.

PROGRAM.

The meetings of the Congress will be held in the Lafayette Square Opera House.

TUESDAY, MAY 1ST.

2:45 P. M.—Congress opened by the Chairman of the Executive Committee.

From 3 P.M. to 5 P.M.—General Session of the Congress. " Subject: " Bacteriology in Health and Disease."

Papers will be read as follows, viz.:

Professor Theobald Smith, of Boston, Mass.: " Adaptation of Pathogenic Bacteria to Different Species of Animals."

Dr. Samuel J. Meltzer, of New York City: " The Physiological Resources of the Body in its Defense against Bacteria and their Toxic Products."

Professor Harold C. Ernst, of Boston, Mass.: " Flagella and Serum Reactions."

Dr. Richard C. Cabot, of Boston, Mass.: " Relation of Bacteriology to Clinical Medicine."

Dr. Edward R. Baldwin, of Saranac, New York: "Bacterio-Therapeutics with Especial Reference to Tuberculosis."

Professor William S. Thayer, of Baltimore, Md.: " The Etiology of Malarial Fevers."

Prof. George Dock, of Ann Arbor, Michigan: " Infection by Animal Parasites."

Professor Simon Flexner, of Philadelphia, Pa.: " Bacteriology of Dysentery."

### WEDNESDAY, MAY 2D.

From 3 to 5 P. M.—General Session of the Congress.

Paper by Professor William Osler, M.D., LL.D., of Baltimore, Md.: " On Modern Therapeutics."

Essay by Dr. Clarence J. Blake, of Boston, Mass.: " Sociological Status of the Physician."

Poem by Dr. S. Weir Mitchell, M.D., LL.D., of Philadelphia, Pa.: " The Evolution of the Physician."

8 P. M.—Address by the President of the Congress, Professor Henry P. Bowditch, M.D., LL.D., D.Sc., Professor of Physiology in the Harvard Medical School, on " The Medical School of the Future."

To be followed by a reception.

### THURSDAY, MAY 3D.

9 P. M.—Banquet for the members of the Congress.

